# Is There a General Factor of Spiritual Intelligence? Factorial Validity of the Polish Adaptation of Spiritual Intelligence Self-Report Inventory

**DOI:** 10.1007/s10943-021-01350-2

**Published:** 2021-07-21

**Authors:** Paweł A. Atroszko, Katarzyna Skrzypińska, Julia M. Balcerowska

**Affiliations:** Institute of Psychology, University of Gdańsk, Bażyńskiego 4, 80-312 Gdańsk, Poland

**Keywords:** Factor analysis, Measurement, Spirituality, Statistics, Validity

## Abstract

In recent years, spirituality and the meaning of life are becoming increasingly important variables in the study of well-being, health, and happiness. The concept of spiritual intelligence (SI) was suggested as a potentially significant construct expanding our understanding of psychological determinants of human functioning. The aim of this paper was to investigate the factorial validity of the Spiritual Intelligence Self-Report Inventory (SISRI; King, 2008) in the context of research on a general factor of spiritual intelligence as a psychological construct. The SISRI was administered to 833 adults in Poland. A four-factor solution with one second-order factor of spiritual intelligence provided an inadequate solution. A four-factor solution with correlated factors and a reduced number of items provided an adequate fit to the data. It is concluded that so far, no data are supporting a single factor of SI measured by SISRI-24, and previous studies, including the original study, show that the measurement with this scale is highly problematic. Without a strong theory and proper measurement, the development of this highly promising area of research may be hindered.

## Introduction

Spirituality as a theoretical psychological construct is still poorly understood, although efforts are made to define and study it (Hood et al., 2009; Oman, 2013; Skrzypińska, 2014). Its nature is widely investigated, especially in relation to the meaning of life (Park, 2005), coping (Pargament, 1997), well-being (Emmons, 1999), and health (Koenig, 2011). Spirituality is reemerging as a vital dimension in the studies on well-being with dynamic developments in the research on mindfulness (Goldberg et al., 2018) and psychedelics (Griffiths et al., 2008), which are areas of research with a reasonably high level of methodological rigor and high-quality findings. Both spiritual traditions and psychological literature (James, 1904; Jung, 2014) point to consciousness transformations and meaning generation as factors in spiritual development (see Frankl, 1966; Zohar & Marshall, 2000; Park, 2005; Skrzypińska, 2014; Mróz et al., 2020). In this context, some have argued that an individual faculty with properties of intelligence may be related to these processes. It was dubbed spiritual intelligence and was postulated to facilitate or enable both consciousness transformations and the creation of personal meaning (SI; Emmons, 1999; Mayer, 2000; Noble, 2000; Skrzypińska, 2020).

### Spirituality, Religiosity, and Meaning-Making

Both the spiritual and religious spheres of the individual have a common denominator: searching and creating the meaning/purpose of life. They are even called sense-making spheres. For example, religion as a meaning-making framework in coping with life stress was described by Park (2005). The author introduced the meaning-making model (MMM), which presents two levels of meaning: systems of global meaning, and the appraised meaning of specific events. Global meaning includes global beliefs and global goals. The latter are described as “internal cognitive structures that individuals construct about the nature of the world” (p. 709). Park (2013) also characterizes spirituality as informing all aspects of global meaning (for example, the human and God’s nature, destiny, karma). This phenomenon provides “ultimate motivation and primary goals for living and special guidelines for achieving these goals with a deep sense of purpose and mattering” (p. 42). This line of reasoning presents the relation between spirituality, religiosity, and the search for meaning/purpose of life. On the other hand, Skrzypińska (2014, 2016), using a theoretical and empirical approach, hypothesized that spirituality is a mediator between the individual view of the world and religiousness. This assumption found some evidence in empirical research. Therefore, it seems that these three phenomena could have a common genesis and, simultaneously, give meaning/purpose to life. Additionally, this complex picture may be complemented by spiritual intelligence, which can be treated as a tool for discovering life's meaning (Emmons, 1999).

### The Aim of the Paper

This paper aims to investigate whether the Polish adaptation of one of the most widely used measures of spiritual intelligence, the Spiritual Intelligence Self-Report Inventory (SISRI; King, 2008), validly measures the general factor of SI with four sub-factors. Valid measurement is the first step between theory and systematic empirical investigation of a phenomenon. Therefore, it is indispensable to show that the construct's measurement is congruent with its theory (Kapuscinski & Masters, 2010). Without a measurement tool, the construct of SI cannot be meaningfully investigated in quantitative studies. Such a lack will considerably limit our understanding of this dimension of human functioning. Notably, lack of a valid SI scale will prevent us from (1) estimating the distribution of the levels of SI in the population, (2) investigating its relationship with other variables including antecedences and consequences, (3) conducting intervention studies aiming at the improvement of SI and evaluating whether it is even possible. If the available scales are not valid, then these particular objectives as well as other related to our knowledge about SI will be impossible to achieve. The scale that lacks validity will yield meaningless and confusing results, only increasing conceptual and empirical bafflement.

Furthermore, it will negatively affect the whole area of research as it will undermine its credibility. Over a century ago, William James postulated systematic and rigorous study of human religious and spiritual experience, among others showing its variety. In the twentieth century, psychological measurement theory underwent remarkable advancements, and currently, it is still being developed. If we want to improve the quality of research in this area, we have to take advantage of the scientific methodology's progress. This way, we may systematically study the range of human spiritual capabilities. One of the most important postulates in scientific inquiry is that we should be able to predict certain empirical outcomes based on theoretical assumptions (Wagenmakers et al., 2012). It means that the SI measurement has to be based on a strong theory of the construct and should not be derived ad hoc from the data. It also means that the data should not be arbitrarily adjusted to the theory.

### Spiritual Intelligence: Conceptualization and Measurement

Spiritual intelligence was suggested as a form of intelligence that concerns a set of capacities and abilities that enable people to solve problems and attain goals in their everyday lives (Emmons, 1999, 2000a, 2000b). This definition is based on the assumption that spirituality may be conceptualized in adaptive, cognitive-motivational terms. In line with this, it has been suggested that SI consists of many abilities and competencies that may be part of a person's expert knowledge relevant to problem-solving situations. Although Emmons (1999) claimed that SI meets Gardner's criteria for independent intelligence in the theory of multiple intelligences (Gardner, 1993), this solution has not been accepted by the author of the theory (Gardner, 2000). What is more, existential intelligence was suggested as feasible (Gardner, 1993, 1999). However, it was not included in the model due to the lack of quantifiable scientific criteria (Gardner, 2000). Both theoretical and practical limitations make SI a highly controversial construct in psychological literature. Among the theoretical limitations, the lack of widely accepted definitions of spirituality and consciousness are the main hindrances (Oman, 2013; Skrzypińska, 2014, 2020; Streib & Hood, 2016).

Nevertheless, it does not deter authors from suggesting new definitions of spiritual intelligence or developing measurement tools. One of the first researchers to take up this ambitious challenge was King (2008). He defined SI as "a set of mental capacities which contribute to the awareness, integration, and adaptive application of the nonmaterial and transcendent aspects of one's existence, leading to such outcomes as deep existential reflection, enhancement of meaning, recognition of a transcendent self, and mastery of spiritual states" (p. 56). The Spiritual Intelligence Self-Report Inventory (SISRI-24) is a scale based on this definition and four core components of spiritual intelligence: critical existential thinking, personal meaning production, transcendental awareness, and conscious state expansion (King, 2008).

Critical existential thinking (CET) means the capacity to contemplate meaning, purpose critically, and other existential/metaphysical issues (e.g., existence, reality, death, the universe); to come to original existential conclusions or philosophies; and to contemplate non-existential issues concerning one's existence (i.e., from an existential perspective). Personal meaning production (PMP) concerns the ability to derive personal meaning and purpose from all physical and mental experiences, including the capacity to create and master (i.e., live according to) a life purpose. Transcendental awareness (TA) refers to the capacity to identify transcendent dimensions/patterns of the self (i.e., a transpersonal or transcendent self), of others, and the physical world (e.g., holism, non-materialism) during normal states of consciousness, accompanied by the capacity to identify their relationship to one's self and the physical world. Conscious state expansion (CSE) is the ability to enter and exit higher/spiritual states of consciousness (e.g., pure consciousness, cosmic consciousness, unity, oneness) at one's discretion (as in deep contemplation or reflection, meditation, prayer, etc.).

### Spiritual Intelligence Self-Report Inventory (SISRI-24): Psychometric Issues

The development of the original scale did not follow the assumptions, guaranteeing meaningful validation of the scale (King, 2008). The initial studies were exploratory primarily. However, the results of the initial exploratory factor analysis yielded six factors, not the assumed four. In this situation, the author arbitrarily removed other factors. According to the author, one factor was removed because it was composed of all 12 reverse-coded items, suggesting "that these items were unstable" (p. 129). Reverse coding is a methodological problem with nothing to do with stability (Lindwall et al., 2012). Removing the items is a practical solution; however, this case shows that a non-confirmatory approach paired with inadequate statistical solutions creates confusing results and methodological artifacts whose effect on the overall model is undetermined. The author removed all reversed coded items, but one was arbitrarily retained. The other factor was removed because the items "collectively had no theoretical basis" (p. 129). In such a situation, one should ask why the original item pool contained items not linked to the SI theory and which were not defined as filler items (that not measure the construct but are used to control random responses from participants) from the start?

Furthermore, multiple items had significant cross-loadings, meaning that they measured more than one factor and (some of them) were subsequently removed. The author noticed that "some factors displayed loadings which did not have any theoretical connection to the other loadings on the same factor (i.e., the theory would have posited these items on other factors)" (p. 130). It casts substantial doubts on the initial operationalization process. At the same time, the author concluded: "In general, the hypothesized factor structure was very well-supported in the initial 84-item pool" (p. 129).

The description of the process of generating the original item pool is very vague. It does not mention any expert panel evaluating content validity or face validity of the items describing assumed theoretical components (only readability testing among 18 adults). The overall results of initial exploratory testing seem to be a simple and unfortunate consequence of that, creating informational noise and a very confusing picture. The subsequent methodological process shows arbitrary cutting out of empirical findings that do not fit the assumed model. It is not only not recommended, but it constitutes a major methodological flaw. The subsequent steps are no less problematic. For example, three additional items were created and added to the second version of the SISRI. It shows that the measurement is not fully derived from the theory but is molded ad hoc based on data. Finally, although the theory assumes one general factor of SI, the original version of the scale tested a model of four correlated factors, never showing that they measure one higher-order factor of SI (King, 2008; King & deCicco, 2009).

To our knowledge, the same is true for all of the subsequent adaptations. Antunes et al. (2018) conducted an exploratory factor analysis on Portuguese adaptation, which showed a factorial structure different from the original scale, comprising three factors instead of four. Transcendental Awareness was removed. In their Chinese adaptation, Chan and Siu (2016) deleted two items after initial confirmatory factor analysis (CFA) because they had nonsignificant loadings. Subsequent CFA on the same dataset (which cannot be considered cross-validation) with the remaining 22 items showed a lack of model fit. It clearly shows that the initial problematic development process resulted in a lack of any replication of the results in the subsequent adaptations.

#### **Hypothesis**

Based on the assumptions behind the SI theory, it is hypothesized that SISRI-24 has a hierarchical structure with four first-order factors loading on one second-order factor of general SI.

## Methods

### Sample

The data were gathered in four different samples, each related to investigating different aspects of concurrent validity (data not presented in this paper). The overall sample comprised 833 participants. Table 1 presents descriptive data on the total sample and the respective samples. The sample size was calculated based on the requirements for conducting CFA in such a relatively complex model (Wolf et al., 2013). The gathered sample allowed for adequate statistical power. It was a cross-sectional study based on convenience sampling. In terms of inclusion criteria, adults of all religions and denominations, agnostic or atheists, willing to participate and approached via procedures described below were included in the study.

**Table 1:** Descriptive data on the samples

Sample	*n*	Gender	Age	Religion^a^
1	183	55.2% women	*M* = 24.50 (SD = 10.04)	69.6%/27.8%/2.5%
2	258	64.9% women	*M* = 28.14 (SD = 9.50)	43.4%/28.3%/28.3%
3	196	69.4% women	*M* = 28.30 (SD = 8.27)	71.1%/21.1%/7.9%
4	196	52.6% women	*M* = 42.35 (SD = 11.74)	88.2%/11.7%/0%
Full sample	833	61.0% women	*M* = 32.23 (SD = 12.70)	76.8%/19.7%/3.5%

^a^Christians/atheists, agnostics or no declaration/other religions

### Instruments

#### Demographics

Questions about age and gender (0 = female, 1 = male), and highest completed education (1 = elementary school, 2 = vocational school, 3 = high school, 4 = Bachelor’s degree, 5 = Master’s degree, 6 = postgraduate studies) were asked. Participants were also asked to endorse one of several response alternatives regarding religion (Catholic, Protestant, Lutheran, Baptist, Presbyterian, Unitarian, Methodist, Mormon, other Christian, Jew, Buddhist, Hindu, Muslim, other religious tradition, Agnostic, Atheist). Data on religion were recoded into three categories: Christians, atheists/agnostics, or no declaration/other religions.

#### Spiritual Intelligence

The Spiritual Intelligence Self-Report Inventory (SISRI-24) assessed spiritual intelligence (King, 2008). It includes 24 items that are grouped into four subscales: critical existential thinking (CET) (an example of the item: *I have spent time contemplating the purpose or reason for my existence*), personal meaning production (PMP) (e.g., *When I experience a failure, I am still able to find meaning in it*), transcendental awareness (TA) (e.g., *I recognize aspects of myself that are deeper than my physical body*), and conscious state expansion (CSE) (e.g., *I can control when I enter higher states of consciousness or awareness*) that represent four components of spiritual intelligence. Respondents provided answers on 5-point Likert scales, ranging from *not at all true of me* (0) to *completely true of me* (4).

### Procedure

This study is a part of a larger, international project called TNS (Threefold Nature of Spirituality; theoretical frames: Skrzypińska, 2014, 2020; international empirical research with an intercultural comparison: Skrzypińska et al., 2019) in which the relationship between spirituality, personality, and human intelligence will be examined. The Spiritual Intelligence Self-Report Inventory (SISRI) was translated in a multistep procedure assuring linguistic equivalence of the Polish version. The SISRI was first translated from English to Polish and then back-translated. After adjustments, the final Polish version was prepared and pretested among bilingual English language students at the Faculty of Philology at the University of Gdańsk. Half of the students (*n* = 33) first filled in the Polish version of the scale, and a half (*n* = 33) filled in the English version of the scale. After two weeks, the first group filled in the English version of the scale, and the second group filled in the Polish version. This procedure was designed to identify significant linguistic problems with particular items. The results suggested good linguistic equivalence of Polish and English versions.

Data collection used convenience sampling. Participants were invited to an anonymous study via Qualtrics platform (subsamples 1 and 4) and GoogleDocs (subsamples 2 and 3) and asked to send the link to the study to their friends, family members, colleagues, and other persons (using "snowball" technique). The study was directed to adults (age 18–65). The estimated response rate was above 85%. Participation in the survey was voluntary and confidential and that participants could withdraw from the study at any time. No monetary or other material rewards were given for participation. The same research was conducted in the other countries simultaneously within the framework of the TNS project. A cross-cultural comparison of the results among different nationalities will be presented in a separate article.

### Statistical Analysis

#### Factor Analyses

A confirmatory factor analysis (CFA) using AMOS, version 24.0, was used to investigate the goodness of fit of the model. The original hierarchical model consisting of four first-order factors (critical existential thinking, personal meaning production, transcendental awareness, and conscious state expansion) and one second-order factor (spiritual intelligence) of the Spiritual Intelligence Self-Report Inventory (SISRI-24) was tested. Lack of correlation between error terms of the indicators was assumed. The maximum likelihood estimation method was used. CFA of the Polish version of the SISRI-24 gave an inadmissible solution, suggesting that the model did not fit to the data. In line with this, a lack of second-order factor (spiritual intelligence) was assumed. Therefore, in the second model, a four-factor structure, in which all factors (critical existential thinking, personal meaning production, transcendental awareness, and conscious state expansion) are correlated, was assumed. Items that had low factor loading (< 0.40) were eliminated. Modification indices were investigated to improve model fit. Items that had the highest covariance of the error terms with other items were also eliminated. The final model assumed a four-factor solution of the SISRI with no correlation of error terms. The following measures were used to evaluate the fit of the model: χ^2^ divided by degrees of freedom (χ^2^/*df*), comparative fit index (CFI), Tucker-Lewis index (TLI), root-mean-squared error of approximation (RMSEA). Cutoff scores for those indexes for acceptable fit are χ^2^/*df* ≤ 3, CFI ≥ 0.95, TLI ≥ 0.95, RMSEA ≤ 0.06 to 0.08 (Hu & Bentler, 1999; Schreiber et al., 2006).

## Results

### Factor Analysis

**Fig. 1:**
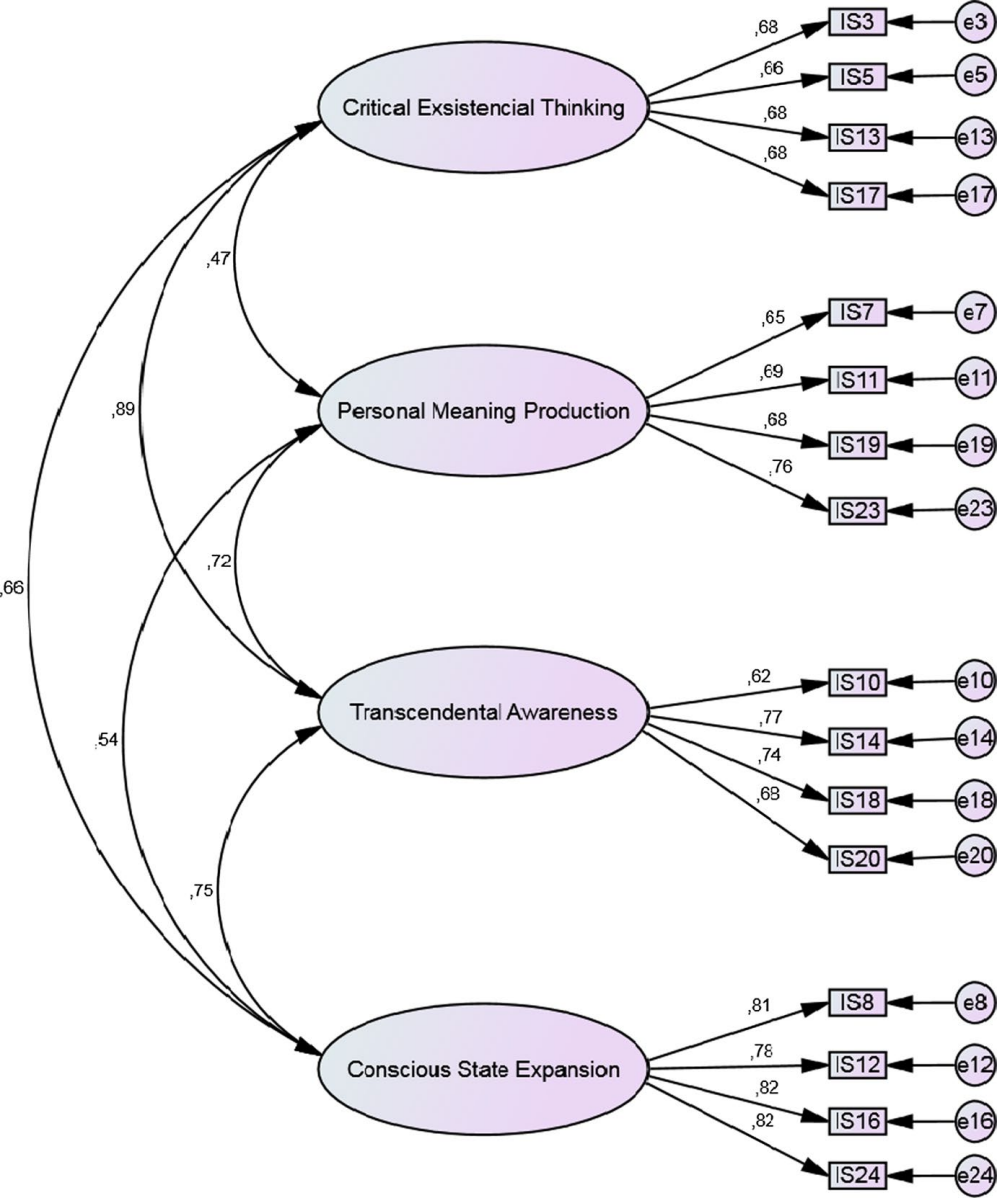
The factor structure and the standardized loadings of the items on the Spiritual Intelligence Self-Report Inventory (SISRI-16)

A four first-order factors and one second-order factor model gave inadmissible solution, suggesting that the model did not fit to the data. Therefore, a four-factor solution in which all factors are correlated was investigated. The second model had a mediocre fit (minimum value of the discrepancy function divided by degrees of freedom [CMIN/DF] = 5.73, root-mean-square error of approximation [RMSEA] = 0.075 (90% confidence interval [CI] 0.072–0.079), comparative fit index [CFI] = 0.88, Tucker-Lewis index [TLI] = 0.86) (Hu & Bentler, 1999). The correlations between factors ranged from 0.44 to 0.83, and the standardized regression weights ranged from 0.31 to 0.83. Due to low factor loading (< 0.40), item 6 was eliminated. On the basis of modification indices, items with highest covariances of residuals with other items (1, 2, 4, 9, 15, 21, 22) were eliminated. The modified model had a good fit: CMIN/DF = 4.56, RMSEA = 0.065 (90% CI 0.059–0.072), CFI = 0.94, TLI = 0.92. The correlations between factors ranged from 0.44 to 0.83 and the standardized regression weights ranged from 0.47 to 0.89 (see Fig. 1).

In the present sample, the Cronbach's alpha reliability coefficient was 0.77 for critical existential thinking, 0.78 for personal meaning production, 0.80 for transcendental awareness, and 0.88 for conscious state expansion. Since the CFA did not support one higher-order factor of SISRI, it is not meaningful to calculate the Cronbach alpha for the general score on the scale.

## Discussion

Based on the assumptions behind the SI theory (Emmons, 1999; King, 2008), it was hypothesized that SISRI-24 has a hierarchical structure with four first-order factors loading on one second-order factor of general SI. The results did not confirm that hypothesis. Instead, a model with a reduced number of items and correlated factors had an acceptable fit to the data. Problems with loadings of particular items were previously found in other validation studies, which also did not replicate the original structure. One study found only three factors (Antunes et al., 2018). Chinese adaptation study found the four correlated factors structure with inadequate fit to the data (Chan & Siu, 2016), similarly to our findings. This study, however, did not additionally investigate the fit by further reducing the problematic items. By eliminating the problematic items, we managed to obtain an adequate fit to the data of the four correlated factors model. However, it has to be emphasized that, at present, this rather constitutes a further molding process of fitting data into the model rather than adjusting the model according to the empirical findings. It should not be concluded based on these results that there are four correlated factors in some way related to spiritual intelligence. It only shows that we may fit the data into such an assumption with a specific analytical process. It also suggests that perhaps with correct operationalization and measurement, it is possible to obtain such a structure. On the other hand, Antunes et al. (2018) found only three factors of SISRI. Therefore, it is more likely that even the result that we obtained in our sample may be just an artifact of the inadequate overall methodological process of fitting data into theoretical assumptions.

These repeatedly found problems with replicating the original structure of SISRI could be likely explained by the process of development of the original scale. It was highly problematic and based on an inadequate methodological approach. The results of the current study, the previous adaptation studies, and the original scale development process suggest that either the theory behind the general SI as postulated by King (2008) needs modification or the operationalization of this theory is inaccurate. Considering all the available data, it is highly recommended to revise the original theory and develop a new tool with a proper methodological approach. Suppose such results concerning factorial structure as the currently and previously reported still appear. In that case, it could indicate that there might not be one faculty akin to intelligence described by such components as King (2008) suggested. It seems likely that these may be correlated but different variables.

These variables might represent or express various human faculties, capacities, beliefs, or values. Moreover, they may even be manifestations of other psychological variables such as narcissism (Vonk & Visser, 2020). Future studies could shed more light on their nature. For example, the preliminary research results indicated that separate scales of SISRI-24 are practically not associated with indicators of rational intelligence (RI), and some of them show weak to medium correlations with emotional intelligence (EI; for TA r-Pearson = 0.31; CSE: *r* = 0.24; PMP *r* = 0.57; Skrzypińska et al., 2019). There is a need to construct a valid and reliable tool for studying SI and compare SI with other types of intelligence, including RI and EI (Skrzypińska, 2020).

### Implications

This study, together with the analyses of the previous research, adds significantly to the existing knowledge on the topic of SI. It provides compelling evidence that SISRI cannot be currently considered a tool that validly measures SI conceptualized as one human faculty with four components. SI is a fascinating problem that may have profound consequences for human well-being. The extraordinary results achieved with spirituality-related interventions such as mindfulness practice (Goldberg et al., 2018) and psychedelics (Griffiths et al., 2008) provide powerful evidence that we may obtain remarkable improvements in well-being and health by modifying human relation to spirituality. These changes could be very difficult or even impossible to achieve with any other means in some cases. However, the theoretical controversies over the nature of the construct of SI (Emmons, 1999, 2000a, 2000b; Gardner, 1993, 1999, 2000) and over the definitions of spirituality itself (Oman, 2013; Skrzypińska, 2014; Streib & Hood, 2016) paired with low-quality empirical research serve considerable disadvantage to the "cause." The current paper may serve as a benchmark and a kind plea (though seemingly harsh criticism) to improve measurement standards in quantitative research on spirituality and particularly on the very promising construct of SI. Spiritually based interventions and spiritual experiences teach us that enlightenment not infrequently starts with a painful confrontation with our limitations. This paper may stimulate improvements in methodological standards in research on SI and may yield benefits to the field.

### Strengths and Limitations

In terms of strengths, a large and varied sample of adult Polish participants was used to test a fairly complex measurement model validly. A major limitation of this study is a nonrepresentative sample, which posits constraints on the generalizability of these results. Perhaps studies on large and more homogenous samples of individuals from particular spiritual/religious backgrounds could shed more light on the scale's psychometric properties. It seems feasible that SISRI-24 might have different validity in different populations, which was previously found, for example, in studies on the measure of mindfulness (Van Dam et al., 2009).

### Conclusions

It is concluded that, to date, no data support a single factor of SI measured by SISRI-24 (King, 2008), and previous studies, including the original study, show that the measurement with this scale is highly problematic. It suggests that either the theory behind SI should be revised and a new tool should be developed, or a more accurate measure based on the existing theory should be created. Perhaps even the legitimacy of the construct itself may need revision. It is possible that SI is not a general ability but rather that what is conceptualized currently as particular components of SI are unique and correlated variables. They may represent or express various human faculties, capacities, beliefs, or values with a significant relationship with psychosocial functioning (Emmons, 1999; King, 2008). Without a strong theory and proper measurement, the development of this up-and-coming area of research may be hindered.

## Appendix: Statements Comprising the SISRI-24 (King, 2008)

**Table:**

No	Statement
1	I have often questioned or pondered the nature of reality
2	I recognize aspects of myself that are deeper than my physical body
3	I have spent time contemplating the purpose or reason for my existence
4	I am able to enter higher states of consciousness or awareness
5	I am able to deeply contemplate what happens after death
6	It is difficult for me to sense anything other than the physical and material*
7	My ability to find meaning and purpose in life helps me adapt to stressful situations
8	I can control when I enter higher states of consciousness or awareness
9	I have developed my own theories about such things as life, death, reality, and existence
10	I am aware of a deeper connection between myself and other people
11	I am able to define a purpose or reason for my life
12	I am able to move freely between levels of consciousness or awareness
13	I frequently contemplate the meaning of events in my life
14	I define myself by my deeper, non-physical self
15	When I experience a failure, I am still able to find meaning in it
16	I often see issues and choices more clearly while in higher states of consciousness/awareness
17	I have often contemplated the relationship between human beings and the rest of the universe
18	I am highly aware of the nonmaterial aspects of life
19	I am able to make decisions according to my purpose in life
20	I recognize qualities in people which are more meaningful than their body, personality, or emotions
21	I have deeply contemplated whether or not there is some greater power or force (e.g., god, goddess, divine being, higher energy, etc.)
22	Recognizing the nonmaterial aspects of life helps me feel centered
23	I am able to find meaning and purpose in my everyday experiences
24	I have developed my own techniques for entering higher states of consciousness or awareness
